# A Rare Case of Stage IV Mixed Neuroendocrine Small Cell and Adenosquamous Cell Carcinoma of the Gallbladder

**DOI:** 10.7759/cureus.26858

**Published:** 2022-07-14

**Authors:** Swetha R Nuthulaganti, Radhika Sharma, Narsimha Candula, Rahul Gujarathi, Jinous Saremian

**Affiliations:** 1 Internal Medicine, University of Florida College of Medicine, Jacksonville, USA; 2 Hospital Medicine, University of Florida College of Medicine, Jacksonville, USA; 3 Pathology, University of Florida College of Medicine, Jacksonville, USA

**Keywords:** computed tomography (ct ), chemotherapy agents, adenosquamous cell carcinoma, neuroendocrine neoplasm, gallbladder

## Abstract

Gallbladder (GB) carcinoma is the fifth most common type of gastrointestinal cancer. Although a majority of these cancers are found to be adenocarcinomas, we present a rare case in which the GB carcinoma was found to have mixed histology with both small cell neuroendocrine carcinoma and adenosquamous cell carcinoma.

## Introduction

Gallbladder carcinomas are the fifth most common type of gastrointestinal carcinoma. Of these tumors, the most common is biliary malignancy [1]. Patients often present with nonspecific symptoms which can range from being asymptomatic to experiencing indigestion and jaundice. Neuroendocrine tumors of the gallbladder account for 2.2% of all gallbladder tumors, while adenosquamous gallbladder carcinoma is noted to be 1% to 12% of all gallbladder carcinomas [2,3]. Both cancers have increased morbidity and mortality. The mainstay of treatment is radical surgery with the addition of adjuvant chemotherapy.

## Case presentation

The patient is a 42-year-old male with no known past medical history who presented with severe right upper quadrant abdominal pain associated with several bouts of diarrhea a day and a significant 20 lbs weight loss over the past six months. The patient stated that his symptoms had significantly worsened over the past few months prompting his presentation to the emergency room. He denied any hematochezia, melena, or discolored stools. At presentation, the patient was hemodynamically stable. Physical exam was notable for right upper quadrant tenderness upon deep palpation. The laboratory profile was normal except for aspartate transaminase (AST) of 61 u/L and alkaline phosphatase (ALP) of 695 u/L.

**Figure 1:**
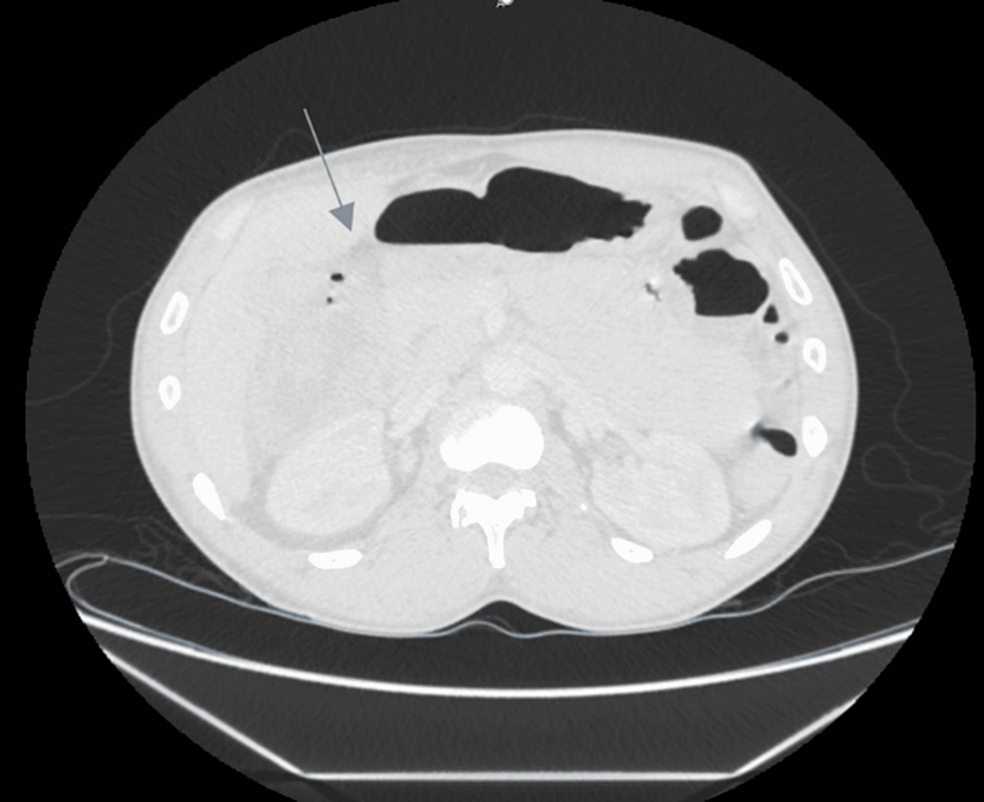
CT of the abdomen and pelvis Small bowel dilatation with the arrow pointing to the transition point

**Figure 2:**
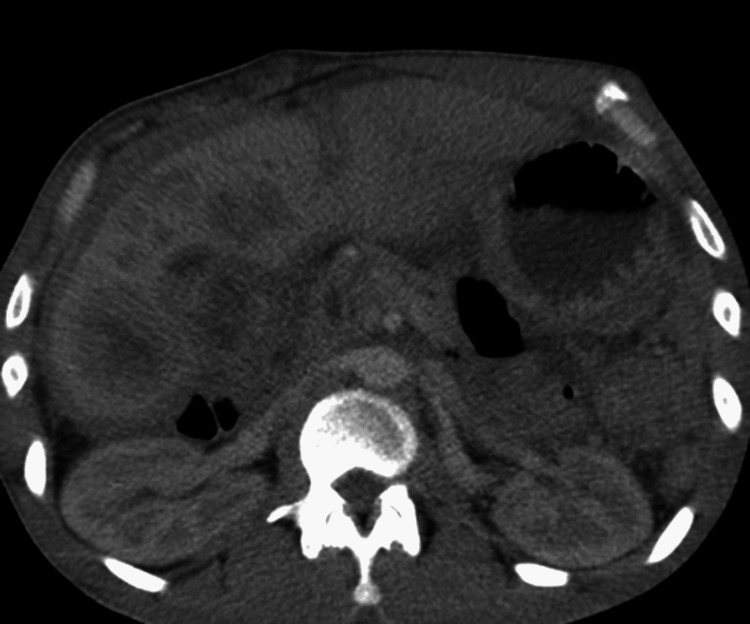
CT of the abdomen and pelvis demonstrating gallbladder mass extending into the lower margin of the liver and invading the duodenum, right hepatic flexure, and pancreatic head; extensive peritoneal carcinomatosis.

**Figure 3:**
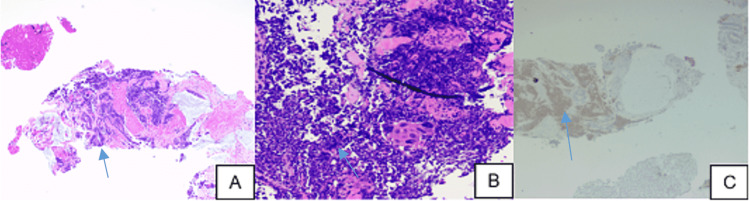
Histology of gallbladder biopsy A: Low power view demonstrating tumor cells as atypical glandular, squamous, and nested with a sheet-like arrangement of cells suggestive of a neuroendocrine component (20X H&E) B: Higher power of neuroendocrine component demonstrating mitosis and apoptosis. Focal squamous differentiation is also seen. (200x H&E) C: Neuroendocrine cells expressing synaptophysin (brown), atypical glandular and squamous components are negative (synaptophysin IHC – 40X) H&E: Hematoxylin and eosin stain

**Figure 4:**
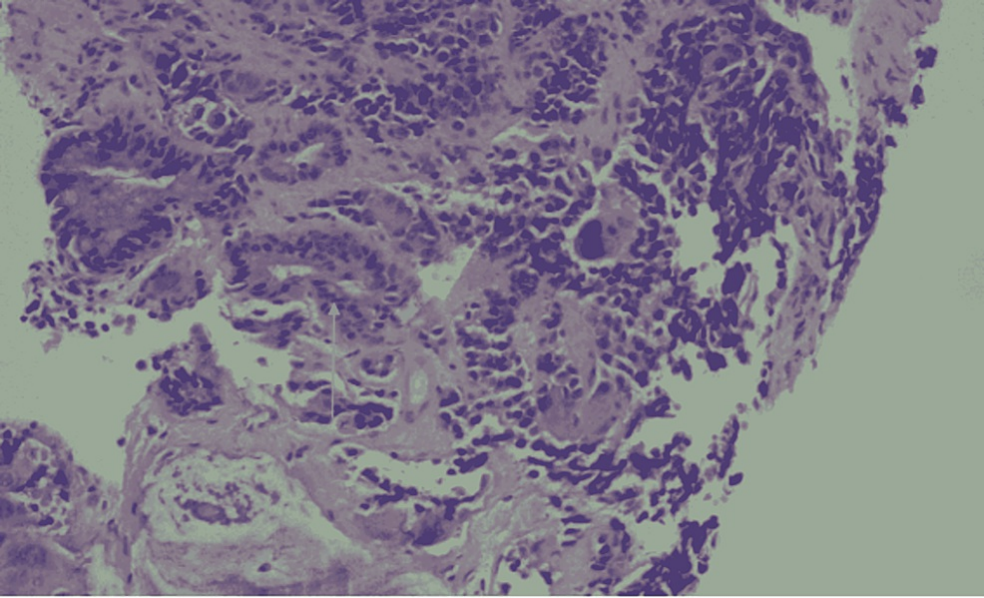
Low power view showing tumor cells in atypical glandular, squamous  and nested and sheet-like (neuroendocrine component) arrangement of tumor cells (20X H&E) In this image, you can see the squamous and glandular components and some areas with neuroendocrine differentiation in the background. H&E: Hematoxylin and eosin stain

Computed tomography (CT) of the abdomen/pelvis revealed nonspecific wall thickening involving the transverse colon extending from the hepatic flexure to the mid-transverse colon concerning for a neoplastic versus inflammatory process (Figure 1 and Figure 2). Irregular mass-like thickening of the gall bladder was also noted. The patient’s CT also demonstrated concerns for complete small bowel obstruction and a diverting ileostomy was performed. A CT-guided needle biopsy of the gallbladder mass demonstrated poorly differentiated mixed neuroendocrine as well as adenosquamous cell cancer (Figure 3 and Figure 4).

Staging CT chest abdomen pelvis demonstrated trace left pleural effusion, pleural metastasis, dilated common bile duct, and gallbladder fossa neoplasm with extensive invasion into the liver parenchyma with metastatic disease throughout the liver. Diffuse intraperitoneal complex ascites consistent with peritoneal carcinomatosis were noted. Peritoneal biopsy was performed which demonstrated metastatic poorly differentiated adenosquamous carcinoma, no neuroendocrine component was seen. The patient was diagnosed with poorly differentiated adenosquamous cell carcinoma of the gallbladder with metastasis to the peritoneum, stage IV, and neuroendocrine small cell cancer of the gallbladder. 

A peripherally inserted central catheter (PICC) line was placed and he was started on chemotherapy with cisplatin, etoposide, and atezolizumab. The patient was discharged with follow-up with hematology and oncology in a week. A few months later the patient decided to pursue hospice care and passed away. 

## Discussion

Most cancers of the gallbladder have been noted to be adenocarcinoma. In particular, adenosquamous cell carcinoma has been associated with a worse prognosis. As a rare cancer subtype, adenosquamous gallbladder carcinoma is noted to be 1% to 12% of all gallbladder carcinomas. Adenosquamous carcinoma is noted to be aggressive with early liver infiltration [2]. The optimal treatment option is radical surgery with the addition of adjuvant chemotherapy. These patients also have increased mortality within two to three months of diagnosis compared to those with other gallbladder carcinomas [4].

Equally rare are neuroendocrine tumors of the gallbladder, which account for 2.2% of all gallbladder tumors [3]. They are more common in women, likely related to the degree of cholelithiasis. These types of tumors are typically found in the gastrointestinal tract and respiratory system; 67% arise in the GI tract and 25% in the respiratory system [3]. The pathogenesis of these tumors is unclear, however, it is postulated that gallbladder neuroendocrine tumors often arise from the epithelial lining of the gallbladder neck. [5]. Argentaffin cells in the metaplastic mucosa are associated with neuroendocrine cell precursors [6]. Neuroendocrine tumor cells have been noted to secrete neurotransmitters, neuromodulators, and neuropeptide hormones. Endocrine cells of this nature typically belong to two functional groups: (1) amine precursor uptake and decarboxylation cells which produce adrenocorticotropic hormone or (2) polypeptide or protein hormones [7]. These tumors are best detected via immunohistochemistry, synaptophysin, and neuron-specific enolase have high specificity. Neuroendocrine tumors are typically categorized by size, angioinvasion, organ invasion, metastasis to the lymph nodes or liver, etc. [8]. Due to its high degree of mortality and lymphatic metastasis, prompt surgical therapy is encouraged. 

Small cell and adenocarcinoma of the gallbladder are noted to result in a locoregional spread that may be secondary to the lymphovascular and peripheral invasion of the gallbladder [9]. As a result, neoadjuvant therapy is not an option due to the early invasion of the cancers; and early surgical resection is recommended. In patients with stage II or less, surgery with cholecystectomy, regional lymphadenectomy, and common bile duct resection may be recommended for curative treatment [10]. Following resection, post-operative chemotherapy within eight to 12 weeks and adjuvant therapy should be offered for six months if the pathological specimen is T2 or higher, node-positive, and margin-positive [10]. For high-grade metastatic tumors, however, medical management is recommended [11]. 

## Conclusions

In the case presented above, our patient demonstrated both adenosquamous cell carcinoma and neuroendocrine small cell of the gallbladder. Both adenosquamous cell carcinoma and neuroendocrine small cell gallbladder are rare forms of gallbladder cancer. However, even more unique is the presence of both histologic features in the pathological specimen sample. We aim to emphasize the early involvement of surgical resection and pathology for curative treatment.
